# Identifying novel genetic variants for brain amyloid deposition: a genome-wide association study in the Korean population

**DOI:** 10.1186/s13195-021-00854-z

**Published:** 2021-06-21

**Authors:** Hang-Rai Kim, Sang-Hyuk Jung, Jaeho Kim, Hyemin Jang, Sung Hoon Kang, Song Hwangbo, Jun Pyo Kim, So Yeon Kim, Beomsu Kim, Soyeon Kim, Jee Hyang Jeong, Soo Jin Yoon, Kyung Won Park, Eun-Joo Kim, Bora Yoon, Jae-Won Jang, Jin Yong Hong, Seong Hye Choi, Young Noh, Ko Woon Kim, Si Eun Kim, Jin San Lee, Na-Yeon Jung, Juyoun Lee, Byeong C. Kim, Sang Joon Son, Chang Hyung Hong, Duk L. Na, Sang Won Seo, Hong-Hee Won, Hee Jin Kim

**Affiliations:** 1 Department of Neurology, Dongguk University Ilsan Hospital, Dongguk University College of Medicine, Goyang, Republic of Korea; 2 Department of Neurology, Samsung Medical Center, Sungkyunkwan University School of Medicine, 81 Irwon-ro, Gangnam-gu, Seoul, 06351 Republic of Korea; 3 Alzheimer’s Disease Convergence Research Center, Samsung Medical Center, Seoul, Republic of Korea; 4 Department of Digital Health, SAIHST, Sungkyunkwan University, Samsung Medical Center, 81 Irwon-ro, Gangnam-gu, Seoul, 06351 Republic of Korea; 5 Department of Biostatistics, Epidemiology and Informatics, Perelman School of Medicine, University of Pennsylvania, Philadelphia, USA; 6 Department of Neurology, Dongtan Sacred Heart Hospital, Hallym University College of Medicine, Hwaseong, Republic of Korea; 7 Department of Neurology, Korea University Guro Hospital, Korea University College of Medicine, Seoul, Korea; 8 Center for Neuroimaging, Radiology and Imaging Sciences, Indiana University School of Medicine, Indianapolis, IN USA; 9 Samsung Genome Institute, Samsung Medical Center, Seoul, Republic of Korea; 10 Department of Neurology, Ewha Womans University Seoul Hospital, Ewha Womans University School of Medicine, Seoul, Republic of Korea; 11 Department of Neurology, Eulji University Hospital, Eulji University School of Medicine, Daejeon, Republic of Korea; 12 Department of Neurology, Dong-A University College of Medicine, Department of Translational Biomedical Sciences, Graduate School of Dong-A University, Busan, Republic of Korea; 13 Department of Neurology, Pusan National University Hospital, Pusan National University School of Medicine and Medical Research Institute, Busan, Republic of Korea; 14 Department of Neurology, Konyang University College of Medicine, Daejeon, Republic of Korea; 15 Department of Neurology, Kangwon National University Hospital, Kangwon National University College of Medicine, Chuncheon, Republic of Korea; 16 Department of Neurology, Yonsei University Wonju College of Medicine, Wonju, Republic of Korea; 17 Department of Neurology, Inha University School of Medicine, Incheon, Republic of Korea; 18 Department of Neurology, Gachon University College of Medicine, Gil Medical Center, Incheon, Republic of Korea; 19 Department of Neurology, School of Medicine, Jeonbuk National University Hospital, Jeonju, Republic of Korea; 20 Department of Neurology, Inje University College of Medicine, Haeundae Paik Hospital, Busan, Republic of Korea; 21 Department of Neurology, Kyung Hee University College of Medicine, Kyung Hee University Hospital, Seoul, Republic of Korea; 22 Department of Neurology, Pusan National University Yangsan Hospital, Pusan National University School of Medicine and Medical Research Institute, Busan, Republic of Korea; 23 Department of Neurology, Chungnam National University Hospital, Daejeon, Republic of Korea; 24 Departmet of Neurology, Chonnam National University School of Medicine, Gwangju, Republic of Korea; 25 Department of Psychiatry, Ajou University School of Medicine, Suwon, Republic of Korea; 26 Department of Health Sciences and Technology, SAIHST, Sungkyunkwan University, Seoul, Republic of Korea; 27 Department of Intelligent Precision Healthcare Convergence, Sungkyunkwan University, Seoul, Republic of Korea

**Keywords:** Alzheimer’s disease, Amyloid-beta, Genome-wide association studies, Positron emission tomography

## Abstract

**Background:**

Genome-wide association studies (GWAS) have identified a number of genetic variants for Alzheimer’s disease (AD). However, most GWAS were conducted in individuals of European ancestry, and non-European populations are still underrepresented in genetic discovery efforts. Here, we performed GWAS to identify single nucleotide polymorphisms (SNPs) associated with amyloid β (Aβ) positivity using a large sample of Korean population.

**Methods:**

One thousand four hundred seventy-four participants of Korean ancestry were recruited from multicenters in South Korea. Discovery dataset consisted of 1190 participants (383 with cognitively unimpaired [CU], 330 with amnestic mild cognitive impairment [aMCI], and 477 with AD dementia [ADD]) and replication dataset consisted of 284 participants (46 with CU, 167 with aMCI, and 71 with ADD). GWAS was conducted to identify SNPs associated with Aβ positivity (measured by amyloid positron emission tomography). Aβ prediction models were developed using the identified SNPs. Furthermore, bioinformatics analysis was conducted for the identified SNPs.

**Results:**

In addition to *APOE*, we identified nine SNPs on chromosome 7, which were associated with a decreased risk of Aβ positivity at a genome-wide suggestive level. Of these nine SNPs, four novel SNPs (rs73375428, rs2903923, rs3828947, and rs11983537) were associated with a decreased risk of Aβ positivity (*p* < 0.05) in the replication dataset. In a meta-analysis, two SNPs (rs7337542 and rs2903923) reached a genome-wide significant level (*p* < 5.0 × 10^−8^). Prediction performance for Aβ positivity increased when rs73375428 were incorporated (area under curve = 0.75; 95% CI = 0.74–0.76) in addition to clinical factors and *APOE* genotype. Cis-eQTL analysis demonstrated that the rs73375428 was associated with decreased expression levels of *FGL2* in the brain.

**Conclusion:**

The novel genetic variants associated with *FGL2* decreased risk of Aβ positivity in the Korean population. This finding may provide a candidate therapeutic target for AD, highlighting the importance of genetic studies in diverse populations.

**Supplementary Information:**

The online version contains supplementary material available at 10.1186/s13195-021-00854-z.

## Background

Genetic factors play an important role in the pathogenesis of Alzheimer’s disease (AD) because heritability is estimated to be 58%–79% [1]. In addition to *APOE* ɛ4, recent genome-wide association studies (GWAS) have discovered a number of genetic risk variants for AD [2, 3]. However, a large proportion of AD heritability is still unexplained.

Accumulation of amyloid-beta (Aβ) in the brain is the earliest pathogenic process in AD, followed by tau deposition, neurodegeneration, and cognitive impairment [4]. Therefore, detecting individuals with Aβ deposition is of utmost importance for the prevention and early treatment of AD [5]. Previous studies have evaluated the genetic basis of Aβ deposition using positron emission tomography (PET) imaging [6–10] and identified several novel Aβ associated genetic variants outside the *APOE* region from European ancestry [11]. However, as each ancestry has a distinct genetic background, replication of the novel genetic findings in different populations is challenging. A number of previous studies failed to replicate European GWAS findings in other ethnic populations [12–15]. Furthermore, it should be noted that most previous GWAS were conducted in individuals of European ancestry, and non-European populations are underrepresented in genetic discovery efforts [16–18].

In this study, using a large sample of the Korean population, we conducted a GWAS to identify single nucleotide polymorphisms (SNPs) associated with Aβ deposition in the brain. We identified novel SNPs for Aβ deposition and demonstrated their associations in an independent cohort of the Korean population. Then, we assessed the topography of Aβ deposition related to the novel SNP. Furthermore, we developed an Aβ prediction model incorporating the novel SNP.

## Materials and methods

### Participants

For the discovery dataset, total 1214 participants of Korean ancestry were recruited from 14 referral hospitals in South Korea from January 2013 to July 2019. Among them, 923 participants were recruited from the Samsung Medical Center, 201 participants were recruited from a multicenter study of the Korean Brain Aging Study for the Early Diagnosis and Prediction of AD (KBASE-V) [19], and 90 participants were recruited from a multicenter study of Clinical Research Platform based on Dementia Cohort.

For the replication dataset, we used data from 306 participants of Korean ancestry from the biobank of the Chronic Cerebrovascular Disease consortium, recruited from 2016 to 2018. This was part of the ongoing Biobank Innovation for chronic Cerebrovascular disease With ALZheimer’s disease Study (BICWALZS) and the Center for Convergence Research of Neurological Disorders.

For the discovery and replication dataset, we included participants (i) who were diagnosed with amnestic mild cognitive impairment (aMCI), AD dementia (ADD), or were cognitively unimpaired (CU) based on detailed neuropsychological tests [20–22], and (ii) who underwent amyloid PET imaging. Participants with aMCI met the following criteria, modified from Peterson’s criteria [23]: (i) normal activities of daily living; (ii) objective memory impairment on verbal or visual memory test, below the 16th percentile of age- and education-matched norms; and (iii) did not have dementia. Those with ADD satisfied the core clinical criteria for probable ADD according to the National Institute of Neurological and Communicative Disorders and Stroke and Alzheimer’s Disease and Related Disorders Association [21]. We excluded participants if they had (i) a causative genetic mutation for AD*,* such as *PSEN1*, *PSEN2*, and *APP*; (ii) structural abnormalities detected on brain MRI, such as severe cerebral ischemia, territorial infarction, or brain tumors; and (iii) other medical or psychiatric diseases that may cause cognitive impairment. All participants provided written informed consent, and the study was approved by the Institutional Review Board of each center.

### Genotyping and imputation

Participants were genotyped using the Illumina Asian Screening Array BeadChip (Illumina, CA, USA) for discovery data and Affymetrix customized Korean chips (Affymetrix, CA, USA) for replication data. Only SNP markers were analyzed. We conducted QC using PLINK software (version 1.9) [24]. Participants were excluded based on the following criteria: (i) call rate < 95%, (ii) mismatch between reported and genetically inferred sex, (iii) deviation from each population parameter, (iv) excess heterozygosity rate (5 standard deviation from the mean), and (v) in cases of related pairs (identified with identity by descent ≥ 0.125) within and between the discovery and replication datasets.

SNPs were excluded based on the following criteria: (i) call rate < 98%, (ii) minor allele frequency (MAF) < 1%, and (iii) a *p* value < 1.0 × 10^−6^ for the Hardy-Weinberg equilibrium test. After QC, genome-wide imputation was performed using the Minimac4 software with all available reference haplotypes from HRC-r1.1 on the University of Michigan Imputation Server [25, 26]. For post-imputation QC, we excluded SNPs based on the following criteria: (i) poor imputation quality (r^2^ ≤ 0.8) and (ii) MAF ≤ 1%. Finally, a total of 4,906,407 SNPs was analyzed.

### Amyloid PET acquisition and image analysis

Amyloid PET images were obtained using a Discovery STE PET/CT scanner (GE Medical Systems, Milwaukee, WI, USA). PET images were acquired for 20 min, starting at 90 min after intravenous injection of either ^18^F-florbetaben or ^18^F-flutemetamol. Aβ positivity or negativity was determined by well-trained nuclear physicians using visual assessments for florbetaben and flutemetamol [27, 28] PET. Briefly, positivity for tracer uptake was assessed in four cortical regions (lateral temporal, frontal, parietal, and posterior cingulate cortices) for florbetaben PET and five cortical regions (lateral temporal, frontal, parietal, posterior cingulate cortices, and striatum) for flutemetamol PET. Amyloid PET positivity was defined as having at least one cortical region with evidence of positive uptake.

A subset of participants in the discovery cohort (*n* = 824) and the replication cohort (*n* = 260) had amyloid PET data available for PET image analysis. For PET image analysis, we performed the following preprocessing using Statistical Parametric Mapping software 12 (SPM, http://www.fil.ion/uc.ac.uk/spm) running on MATLAB (MathWorks 2014b): (1) co-registration of PET to T1-weighted structural MRI, (2) structural MRI segmentation and calculation of transformation matrix, (3) normalization of PET to a Montreal Neurological Institute (MNI) space, and (4) spatial smoothing with a Gaussian kernel of 8-mm full width at half maximum. To calculate the standardized uptake value ratio (SUVR) for each PET image, we used two reference regions (the cerebellar cortex for florbetaben and pons for flutemetamol). The masks of reference regions were obtained from the GAAIN website (http://www.GAAIN.org).

### Statistical analysis

#### GWAS analysis

Logistic regression analysis was performed to determine the association between SNPs and Aβ positivity controlling for age, sex, and the first three principal components (PC) of the genetic ancestry, expressed as Aβ positivity = β_0_ + β_1_ age + β_2_ sex + β_3_ PC_1_ + β_4_ PC_2_ + β_5_ PC_3_ + β_6_ SNP (additive model, coded as 0, 1, and 2 according to the number of minor alleles). Reported *p* values were two-tailed, and we defined a *p* value less than 5.0 × 10^−8^ as being statistically significant and less than 1.0 × 10^−5^ or 1.0 × 10^−6^ as being statistically suggestive based on previous studies [29–31]. We assessed genomic inflation according to a previous study [32]. For the replication analysis, reported *p* values were two-tailed, and a *p* value less than 0.05, was considered statistically significant. Furthermore, considering the small size of the replication dataset, we performed a permutation test to infer the statistical significance of SNPs from the null distribution. We recalculated the t values of SNPs from logistic regression analysis of randomly shuffled Aβ positivity (10,000 permutations). We calculated the fraction of permutations that showed a more significant association than the observed t values of SNPs derived from the original dataset.

To check if SNPs were associated with Aβ positivity independent of *APOE* genotype, we performed a conditional analysis by further adjusting for *APOE* genotype. We also performed a *p* value based meta-analysis and calculated the summary effect size by averaging the study specific effect sizes, with weights reflecting the standard errors from the study specific effect sizes.

#### Effects of the newly identified SNPs

After identifying associated SNPs, we calculated the risk of the identified SNPs on Aβ deposition in all participants and at each cognitive level (CU, aMCI, and ADD). We also examined whether Aβ associated SNPs are associated with ADD risk using CU and ADD participants using the following logistic model: ADD = β_0_ + β_1_ age + β_2_ sex + β_3_ education + β_4_ identified SNPs.

Next, using the previously reported cut-off values for Aβ positivity (SUVR 0.6 for flutemetamol [33], and SUVR 1.4 for florbetaben [34]), we also performed logistic regression to evaluate whether the identified SNPs were associated with Aβ deposition based on SUVR cut-off values.

Furthermore, we performed voxel-wise PET image analysis to determine which regional Aβ deposition is associated with SNPs after adjusting for the effects of age, sex, genetic PCs, *APOE* genotype, and PET tracer type. T static maps were thresholded by *p* < 0.001 with cluster size > 20 when uncorrected for multiple tests or *p* < 0.05 when corrected for multiple tests using family-wise rate.

To test the clinical utility of the newly identified SNPs, we developed multivariable logistic models to predict Aβ positivity in each individual. To evaluate the performance of the logistic model, we measured the area under curve (AUC) from the receiver operating characteristic curve analysis. For internal validation, we conducted a 10-fold cross-validation with 100 repeats using the discovery data. We reported the mean AUC with 95% confidence interval (CI) of the model. As an external validation, parameters estimated from the discovery data were used to test the Aβ prediction performance in the replication data. We used R software (http://www.r-project.org) and MATLAB for the statistical analyses and results visualization.

Finally, we characterized the function of the identified SNPs by leveraging bioinformatic tools and previously reported results. First, we checked whether MAF of SNPs in our data was similar to that in the East Asian population using the 1000 Genomes Project dataset [35]. To evaluate the genotype-specific expression of identified SNPs in human brain tissues, we performed cis-expression quantitative trait loci (cis-eQTL) analysis through the Genotype-Tissue Expression portal (https://gtexportal.org) [36]. We reported genes that showed significant expression changes in the brain tissues (*p* < 0.05).

## Results

### Participants

After QC of genotype data, a total of 1190 (383 CU, 330 aMCI, and 477 ADD) and 284 participants (46 CU, 167 aMCI, and 71 ADD) remained available for the discovery and replication data, respectively. Table 1 shows the baseline demographics for the two datasets (discovery and replication data).

**Table 1:** Demographics of study participants

	Discovery data				Replication data				
Demographics	Total (***n*** = 1190)	Aβ negative (***n*** = 561)	Aβ positive (***n*** = 629)	***p***†	Total (***n*** = 284)	Aβ negative (***n*** = 180)	Aβ positive (***n*** = 104)	***p***^**†**^	***p***^**††**^
**Age, year (SD)**	70.07 (8.75)	70.06 (8.16)	70.07 (9.25)	0.990	72.67 (7.32)	71.76 (7.31)	74.25 (7.10)	0.006	< 0.001
**Female, n (%)**	680 (57.1)	310 (55.3)	370 (58.8)	0.215	184 (64.8)	122 (67.8)	62 (59.6)	0.165	0.019
**Education, year (SD)**	11.02 (4.86)	10.89 (5.04)	11.13 (4.70)	0.390	8.34 (5.21)	7.77 (5.19)	9.11 (5.15)	0.050	< 0.001
**Diagnosis, n (%)**									
**CU**	383 (32.2)	326 (58.1)	57 (9.1)	< 0.001	46 (16.2)	43 (23.9)	3 (2.9)	< 0.001	< 0.001
**aMCI**	330 (27.7)	172 (30.7)	158 (25.1)		167 (58.8)	125 (69.4)	42 (40.4)		
**ADD**	477 (40.1)	63 (11.2)	414 (65.8)		71 (25.0)	12 (6.7)	59 (56.7)		

^†^*P* value was calculated by comparing Aβ negative and Aβ positive participants. ^††^*P* value was calculated by comparing discovery data and replication data. Student’s t test and chi-squared test were used for continuous and categorical variables, respectively.

*Abbreviations*: *Aβ* amyloid β, *ADD* Alzheimer’s disease dementia, *aMCI* amnestic mild cognitive impairment, *CU* cognitive unimpaired, *SD* standard deviation

### GWAS analysis

**Fig. 1:**
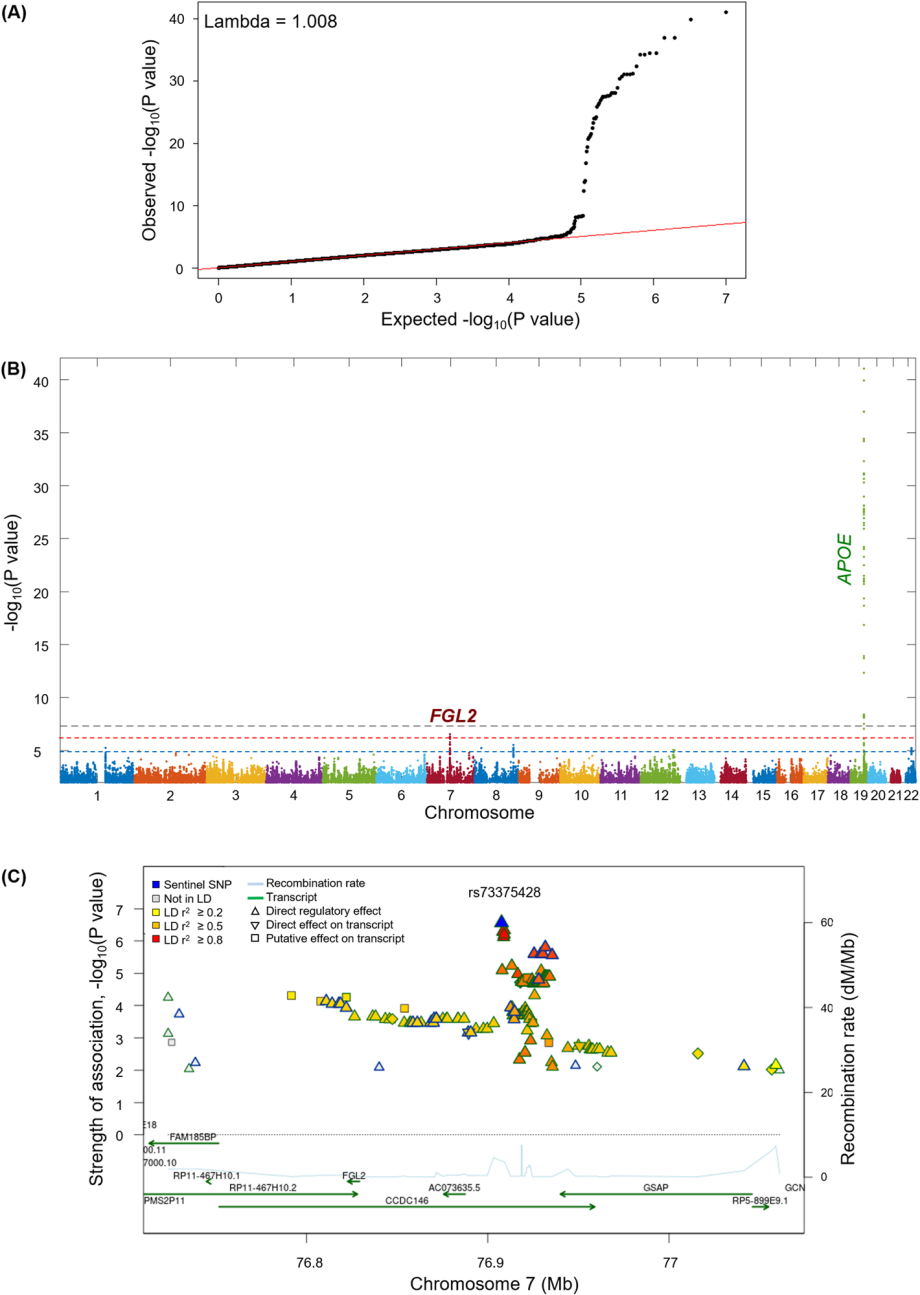
**a** Q-Q plot. **b** Manhattan plot of GWAS analysis. **c** Regional association plot of rs73375428. The dotted line in the Manhattan plot indicate the genome-wide significance level (*p* = 5.0 × 10^−8^, gray dotted line) and the genome-wide suggestive level (*p* = 1.0 × 10^−6^, red dotted line; *p* = 1.0 × 10^−5^, blue dotted line), respectively. Regional association plot was modified from the SNiPA (single nucleotide polymorphism annotator) (https://snipa.helmholtz-muenchen.de/snipa3). GWAS, genome-wide association study; MAF, minor allele frequency; Q-Q plot, quantile-quantile plot; SNP, single nucleotide polymorphism; SPDYE18, speedy/RINGO cell cycle regulator family member E18; PMS2P9, PMS1 homolog2 mismatch repair system component pseudogene 9; FAM185BP, family with sequence similarity 185 member A pseudogene; SPDYE17, speedy/RINGO cell cycle regulator family member E17; 1-UPK3BP1-PMS2P11, uroplakin 3B pseudogene 1- PMS1 homolog2 mismatch repair system component pseudogene11; FGL2, fibrinogen-like protein 2; CCDC146, coiled-coil domain containing 146; GSAP, gamma secretase activating protein; GCNT1P5, glucosaminyl transferase 1 pseudogene 5

A quantile-quantile plot of *p* values revealed no genomic inflation (λ = 1.008) (Fig. 1a). In the discovery data, we identified 61 genome-wide significant SNPs on chromosome 19 (*p* < 5.0 × 10^−8^) (Fig. 1b). However, all significant SNPs fell within the 500 kb region surrounding *APOE* and lost genome-wide significance when we adjusted for the *APOE* ɛ4 allele (Table S1). Outside of the *APOE* region, 38 SNPs on chromosomes 1, 7, 8, 12, and 22 (*p* < 1.0 × 10^−5^), and nine SNPs on chromosome 7 (*<* 1.0 × 10^−6^) showed genome-wide suggestive significance (Table S2). Among the nine SNPs, four were associated with Aβ positivity (*p* < 0.05) in the replication dataset (Table 2). The permutation test of all four SNPs showed t-values lower than the lowest 5% of 10,000 permutations (Table 2, Figure S1).

**Table 2:** Associations between SNPs and Aβ positivity in the two datasets

SNP	EA	Analysis 1							Analysis 2						
		Discovery data		Replication data			Meta-analysis		Discovery data		Replication data			Meta-analysis	
		OR	***p***	OR	***p***	***p***^**†**^	OR	***p***	OR	***p***	OR	***p***	***p***^**†**^	OR	***p***
**rs73375428**	G	0.519	2.71 × 10^−7^	0.550	0.040	0.0163	0.526	3.35 × 10^−8^	0.535	1.23 × 10^−5^	0.481	0.022	0.0101	0.516	8.00 × 10^−7^
**rs2903923**	G	0.529	5.15 × 10^−7^	0.539	0.032	0.0136	0.536	4.97 × 10^−8^	0.546	2.19 × 10^−5^	0.478	0.020	0.0058	0.510	1.32 × 10^−6^
**rs3828947**	C	0.529	5.15 × 10^−7^	0.547	0.036	0.0155	0.536	5.59 × 10^−8^	0.546	2.19 × 10^−5^	0.480	0.020	0.0056	0.515	1.39 × 10^−6^
**rs11983537**	T	0.558	7.58 × 10^−7^	0.539	0.026	0.0127	0.563	5.92 × 10^−8^	0.570	1.99 × 10^−5^	0.492	0.020	0.0091	0.517	1.22 × 10^−6^
**rs112599253**	T	0.561	1.56 × 10^−7^	0.723	0.214				0.586	6.69 × 10^−5^	0.698	0.210			
**rs79761449**	T	0.564	2.50 × 10^−7^	0.723	0.214				0.579	5.22 × 10^−5^	0.698	0.210			
**rs6971106**	T	0.564	2.50 × 10^−7^	0.723	0.214				0.579	5.22 × 10^−5^	0.698	0.210			
**rs6978259**	C	0.522	4.62 × 10^−7^	0.566	0.060				0.521	7.90 × 10^−6^	0.515	0.040			
**rs6958464**	T	0.526	6.28 × 10^−7^	0.555	0.056				0.524	9.63 × 10^−6^	0.484	0.028			

Analysis 1 is a logistic regression analysis, expressed as Aβ positivity = β_0_ + β_1_ age + β_2_ sex + β_3_ PC_1_ + β_4_ PC_2_ + β_5_ PC_3_ + β_6_ SNP

Analysis 2 is a logistic regression analysis, expressed as Aβ positivity = β_0_ + β_1_ age + β_2_ sex + β_3_ PC_1_ + β_4_ PC_2_ + β_5_ PC_3_ + β_6_ *APOE* ɛ4+ β_7_ SNP

^†^*P values* were calculated using permutation tests

*Abbreviations*: *BP* base pair, *C* cytosine, *CHR* chromosome, *EA* effective allele, *OR* odds ratio, *G* guanine, *SNP* single nucleotide polymorphism, *T* thymine

Of the four SNPs, rs11983537 was genotyped while the remaining were imputed. Imputation qualities of the identified SNPs were high (mean *r*^2^ 0.97 ± 0.02). Of note, two of the four SNPs (rs73375428 and rs2903923) showed genome-wide significant associations (*p* < 5.0 × 10^−8^) in the meta-analysis of the discovery and replication datasets (Table 2)*.* When we adjusted for the effect of the *APOE* ɛ4 allele, all four SNPs were associated with Aβ positivity in the replication datasets (*p* < 0.05) (Table 2). Since the identified four SNPs showed high linkage disequilibrium (mean *r*^2^ 0.95 ± 0.05) with each other, we selected rs73375428 for subsequent analyses because it showed the most significant association in the primary analysis of the discovery dataset.

### Effects of the newly identified SNPs

In the logistic model, the *APOE* ɛ4 allele was associated with a 5-fold higher risk of Aβ positivity (odds ratio [OR] = 5.330; 95% CI = 4.188–6.788; *p* < 0.001) and rs73375428 was associated with a 2-fold lower risk of Aβ positivity (OR = 0.519; 95% CI = 0.404–0.666; *p* < 0.001). When we adjusted the effect of diagnosis (CU, aMCI, and ADD), the effect of rs73375428 remained significant (OR = 0.556; 95% CI = 0.406–0.666; *p* < 0.001). In the subgroup analysis, the association of rs73375428 with Aβ positivity was significant in the CU and aMCI groups but not in the ADD group, while the association of *APOE* ɛ4 was significant across all cognitive states (Table 3). When we defined Aβ positivity based on SUVR, rs73375428 was also associated with a decreased risk of Aβ positivity in both discovery (OR = 0.608; 95% CI = 0.523–0.707; *p* < 0.001) and replication (OR = 0.551; 95% CI = 0.408–0.744; *p* = 0.047) datasets (Table S3).

**Table 3:** Risk of having a minor allele in rs73375428 (G) or *APOE* ɛ4 on Aβ positivity

	rs73375428		***APOE*** ɛ4	
	OR (95% CI)	***p***	OR (95% CI)	***p***
**Total (*n*** **= 1190)**	0.519 (0.404–0.666)	< 0.001	5.330 (4.188–6.788)	< 0.001
**CU (*n*** **= 383)**	0.486 (0.244–0.964)	0.030	3.885 (2.307–6.54)	< 0.001
**aMCI (*n*** **= 330)**	0.463 (0.286–0.749)	0.001	6.655 (4.101–10.8)	< 0.001
**ADD (*n*** **= 477)**	0.685 (0.370–1.270)	0.230	4.272 (2.428–7.516)	< 0.001

Logistic regression analysis was adjusted for age and sex

*Abbreviations*: *ADD* Alzheimer’s disease dementia, *aMCI* amnestic mild cognitive impairment, *CU* cognitive unimpaired, *OR* odds ratio

**Fig. 2:**
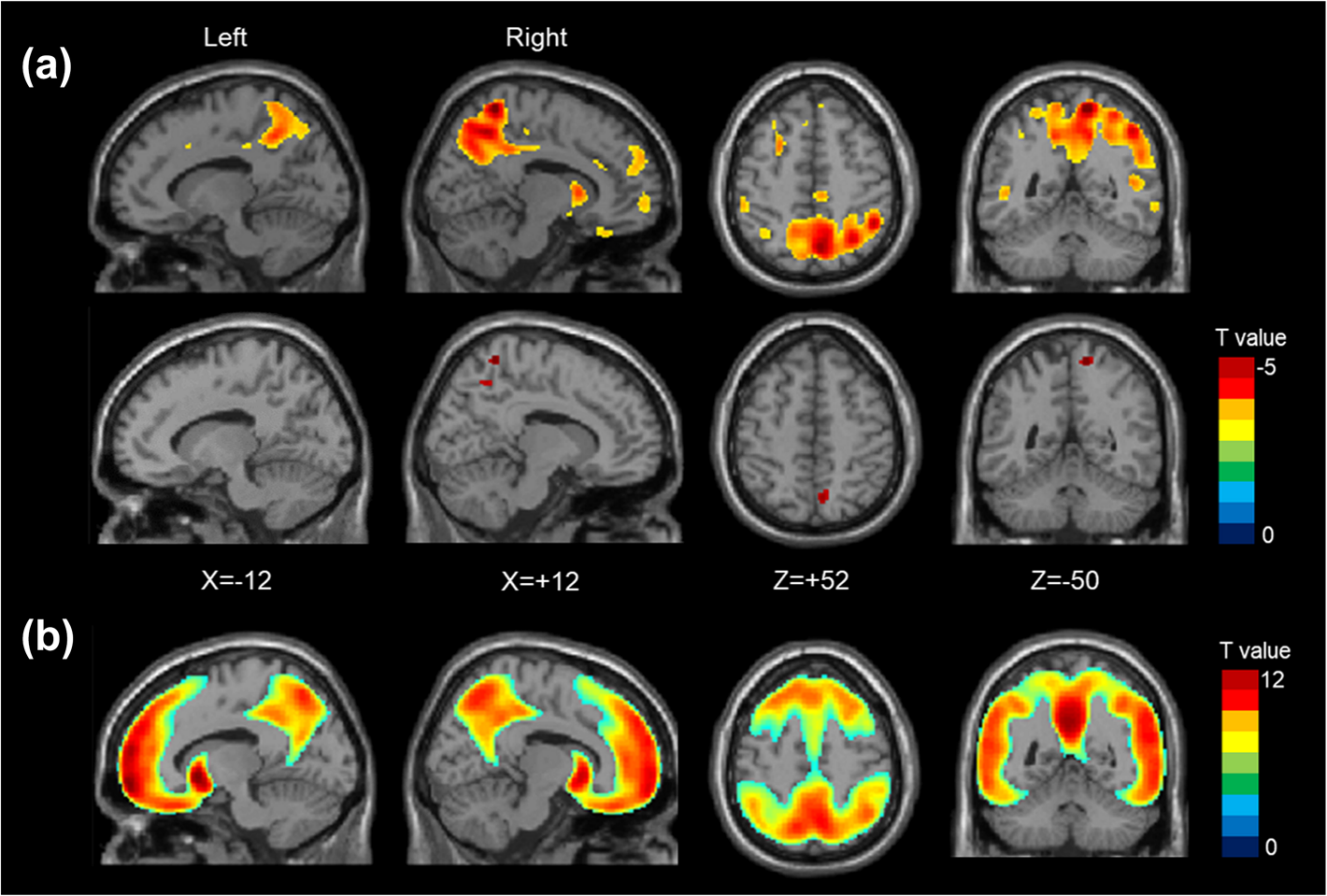
Results of voxel-wise PET image analysis. T static maps showing **a** decreased Aβ deposition in participants with the minor allele of the rs73375428 variant (first row: thresholded by uncorrected *p* < 0.001 with cluster size > 20; second row: thresholded by family-wise rate-corrected *p* < 0.05) and **b** increased Aβ deposition in participants with *APOE* ɛ4 allele (thresholded by family-wise rate corrected *p* < 0.05). X and Z are based on MNI coordinates. Aβ, amyloid β; MNI, Montreal Neurological Institute

In the voxel-wise PET image analysis, *APOE* ɛ4 was associated with increased Aβ deposition on the wide cortical areas of the frontal, parietal, and temporal lobes. The SNP rs73375428 was associated with decreased Aβ deposition in the precuneus, lateral parietal, and medial frontal areas, independent of age, sex, genetic PCs, *APOE* ɛ4, and PET tracer type (Fig. 2).

We additionally analyzed the risk of *APOE* ɛ4 and rs73375428 on the clinical diagnosis of ADD. *APOE* ɛ4 significantly increased ADD risk (OR = 3.413; 95% CI = 2.63–4.42; *p* < 0.001) independent of age, sex, education, and rs73375428; and rs73375428 significantly decreased ADD risk (OR = 0.579; 95% CI = 0.421–0.795; *p* < 0.001) independent of age, sex, education, and *APOE* ɛ4.

**Fig. 3:**
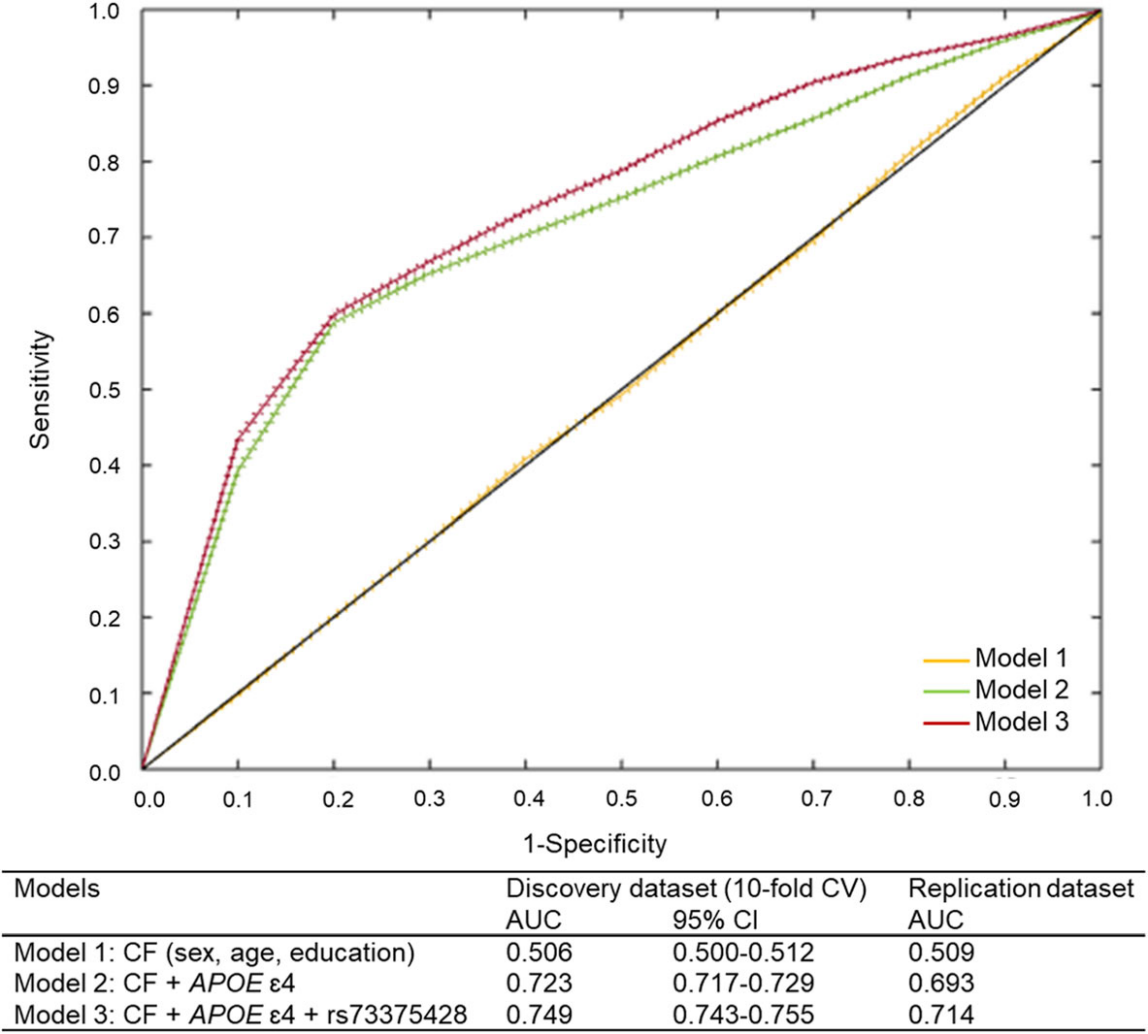
ROC curves for the prediction of Aβ positivity. Solid lines indicate the mean of AUC and dotted lines indicate 95% CIs of AUC. Each model is developed by the multivariate logistic regression. Aβ, amyloid β; AUC, area under curve; CF, clinical factors; ROC, receiver-operating characteristic

We developed prediction models to test the clinical utility of the *APOE* ɛ4 allele and newly identified SNP (rs73375428) in predicting Aβ positivity. In the 10-fold cross-validation with 100 repetitions, the model (model 1) including only clinical factors (age, sex, and level of education) showed an AUC of 0.506 (95% CI = 0.500–0.512). After incorporating the *APOE* ɛ4 allele in the model (model 2), the prediction performance significantly increased (AUC = 0.723; 95% CI = 0.717–0.729). Moreover, when the model included rs73375428 (model 3), the prediction performance further increased (AUC = 0.749; 95% CI = 0.743–0.755) (Fig. 3). When each model, trained in the discovery data, was tested in the replication data, the highest AUC was also observed in the model including both *APOE* ɛ4 and rs73375428 (model 1 AUC = 0.509, model 2 AUC = 0.693, model 3 AUC = 0.714).

### Cis-eQTL analysis

rs73375428 was located in the intron of the coiled-coil domain containing the 146 (*CCDC146*) gene (Fig. 1c). After identifying three additional SNPs with high LD (*r*^2^ > 0.7) (rs11983537, rs6978259, and rs3828947), we performed cis-eQTL analysis using the GTEx database. We found that two SNPs (rs73375428 and rs6978259) had significant cis-eQTL effects on the fibrinogen-like protein 2 (*FGL2*) gene in the brain cortex. Furthermore, a greater dosage of minor allele in SNPs was associated with decreased expression of *FGL2* in the brain cortex (rs73375428, normalized effect size [NES] = -0.175, *p* = 0.02; rs6978259, NES = -0.176, *p* = 0.01).

### Association of previously reported Aβ risk loci from European populations with Aβ positivity in the Korean population

Among the 16 Aβ-associated SNPs reported by Yan et al. [11], no SNP outside the *APOE* region showed significant association with Aβ positivity and only *MAGEF1* (OR = 0.810, *p* = 0.058) locus showed marginal association in our cohort (Table S4). Based on the public dataset (1000 Genomes Project phase 3) [35], the frequency of the previously reported SNPs differed between Europeans and East Asians, while our cohort (Korean) showed similar allele frequencies to that of East Asians (Table S4).

## Discussion

We performed GWAS to identify genetic factors associated with Aβ deposition in the brain using the largest amyloid PET imaging and GWAS data collected from multicenters in South Korea. We identified four novel SNPs (rs73375428, rs2903923, rs3828947, and rs11983537) on chromosome 7, which were associated with a decreased risk of Aβ positivity in the brain at the suggestive level (*<* 1.0 × 10^−6^). These associations were also observed in the independent cohort (*p* < 0.05). Having a minor allele in rs73375428 (G) was associated with a 2-fold decreased risk of Aβ positivity (OR = 0.519) and decreased Aβ deposition in the precuneus, lateral parietal, and medial frontal areas. Incorporating rs73375428, in addition to age, sex, education, and *APOE* e4, better predicted Aβ positivity. The minor allele of rs73375428 was associated with decreased expression levels of *FGL2* in the brain.

We identified four novel SNPs (rs73375428, rs2903923, rs3828947, and rs11983537) associated with a decreased risk of Aβ positivity in the brain. In the discovery dataset, nine SNPs showed genome-wide suggestive significance (*<* 1.0 × 10^−6^), of which four SNPs were associated with a decreased risk of Aβ positivity (*p* < 0.05) in an independent cohort. Although the significance of four novel SNPs was at the suggestive level, meta-analysis of the discovery and replication datasets showed that two SNPs (rs73375428 and rs2903923) reached a genome-wide significance level (*p* < 5.0 × 10^−8^). Furthermore, the obtained OR of rs73375428 for Aβ positivity was 0.519, which was strong compared with the ORs of previously reported Aβ- or ADD-associated SNPs (Aβ-associated SNPs OR from 0.84 to 1.2 [13]). In our cohort, about 30% of CU participants carried one or more minor alleles in rs73375428 (MAF of 0.160). This is in accordance with the previously reported MAF of rs73375428 in the East Asian population (MAF of 0.131) [35], which indicates that the samples used in this study were not biased and may reflect the East Asian population. In the subgroup analysis, the identified SNP (rs73375428) decreased the risk of Aβ positivity in the CU and aMCI group but not in the ADD group. This finding may suggest that in the course of AD spectrum, the effect of rs73375428 diminishes in the dementia stage.

Further imaging analysis and prediction model for Aβ positivity showed consistent results. PET image analysis showed that the participants with minor allele in rs73375428 had less Aβ deposition in the precuneus, lateral parietal, and medial frontal areas. These areas are part of the default mode network, typical regions where Aβ deposits in AD [37]. Identifying patients with Aβ deposition is of the utmost importance in predicting the prognosis and selecting patients for clinical trials of anti-Aβ therapy [38]. Currently available diagnostic tools for measuring Aβ are either invasive (cerebrospinal fluid examination) or expensive (PET), hampering their widespread application in clinical practice [39]. We demonstrated that genetic data (*APOE* ɛ4 and rs73375428) obtained from blood samples with clinical information could predict Aβ positivity with an AUC of 0.749. Furthermore, we demonstrated that the prediction performance improved when rs73375428 was included in the model in addition to age, sex, and *APOE ɛ4*, suggesting the clinical utility of rs73375428.

The identified SNPs were associated with decreased expression of *FGL2* in the brain cortex. Although further specific biological mechanistic studies are required, this result suggests that *FGL2* may be a possible link between rs73375428 and decreased Aβ deposition in the brain. FGL2 is a membrane-bound or secreted protein expressed by immune cells that have either coagulation activity [40, 41] or immune-suppressive functions [42, 43]. A previous study demonstrated that *FGL2* expression is associated with brain tumor progression through the immune system [44]. *FGL2* was also associated with AD. One prior study demonstrated that when human microglia were exposed to Aβ peptide, *FGL2* expression in microglia was reduced more than six-fold as an inflammatory response to Aβ peptide [45]. Furthermore, Taguchi et al. obtained brain samples from both patients with AD and controls of Japanese population and demonstrated that *FGL2* was upregulated in the AD hippocampus as compared to controls [46]. Given these previous observations, we speculated that participants with minor alleles of rs73375428 could have reduced the risk of Aβ deposition in the brain through decreased expression of *FGL2*, which reflects the reactive inflammatory response (e.g., Aβ clearance) to Aβ peptide. More functional studies are necessary to elucidate the role of *FGL2* in AD pathogenesis.

Our results showed some evidence for ethnic similarity and differences in genetic variants associated with Aβ. As expected, variants in the *APOE* locus exhibited a significant association with Aβ deposition in the brain, confirming that the *APOE* variants are important risk factors for AD across various ethnicities [47]. However, there were some ethnic differences. We observed a stronger effect of the variant in *APOE* (rs429358) on Aβ positivity in the Korean population than that in the European population (Korean, OR = 5.275; European, OR = 1.197 [11]). This is similar to the results in previous studies of the East Asian population, in which the effect of *APOE* ɛ4 on AD risk was stronger in Han Chinese [48] and Japanese [47] than in the European population. Furthermore, outside the *APOE* locus, previously reported Aβ associated SNPs in European ancestry data were not replicated [11] in our cohort. Ethnic differences in the effect size and significance might be attributed to the differences in allele frequency and LD pattern across different populations [12]. Indeed, we observed heterogeneity in the allele frequency between the European and Korean cohorts (Table S4). Furthermore, epigenomic patterns, lifestyle, education attainment, and other non-genetic factors may also account for differences across populations. However, it should be noted that the lack of replication might also be a result of insufficient sample size of our cohort. Nevertheless, these findings suggest that the discovery from GWAS in one population may not be applicable to other populations. Therefore, continuous efforts of population-specific and trans-ethnic studies are necessary to accurately discover risk genetic variants.

## Limitations

This study has several limitations. First, the statistical significance of the novel SNP was at the genome-wide suggestive level, and the sample size of the replication dataset was small. Furthermore, although associations between four SNPs and Aβ (*p* < 0.05) were found in the independent dataset, the statistical significance disappeared after correction for multiple tests of nine SNPs. However, our study might present true findings for the following reasons: (i) nine suggestive SNPs at a more conservative *p*-value (< 1.0 × 10^−6^) showed high LD with each other, which might reduce the number of independent tests to one; (ii) the permutation test of the four SNPs showed that if the null hypothesis was true, the chance of observing our findings would be extremely small for a given sample size; (iii) two SNPs (rs73375428 and rs2903923) showed genome-wide significant associations in the meta-analysis; and (iv) the biological relevance of *FGL2* association with the identified SNPs in the brain tissue suggests a potential AD-associated gene. Nevertheless, our findings should be interpreted with caution and replicated in larger independent datasets. Second, imputation was performed using a large reference panel of mixed populations rather than the Korean population. However, we conducted a strict post-imputation QC, excluding SNPs with poor imputation quality (*r*^2^ ≤ 0.8) or low frequency (MAF < 1%). As a result, the imputation qualities of the identified SNPs were high (mean *r*^2^ 0.97 ± 0.02). Third, the cis-eQTL dataset was obtained from healthy populations and not from subjects with AD. Furthermore, the causality of the identified SNPs and *FGL2* expression could not be evaluated in the current analysis. Functional studies using gene editing are necessary to determine the association between the identified SNPs and *FGL2.* Fourth, GWAS was conducted using Aβ positivity, determined by the visual assessment not by quantitative Aβ SUVR. Since this study was conducted using large data obtained from multiple cohorts, some data were not available for SUVR analysis. However, the visual assessment of Aβ positivity has high correlations with histopathological findings of Aβ deposition in the brain [49, 50], and it is more widely used in the clinical practice.

## Conclusions

We identified novel SNPs that reduce the risk of Aβ deposition in the brain and suggested a possible role of *FGL2* in AD pathogenesis. This finding may provide a candidate therapeutic target for AD, highlighting the importance of genetic studies in diverse populations.

## Supplementary Information

**Additional file 1: Table S1.** Significant (*p* value< 5.0×10^-8^) SNPs associated with Aβ positivity. **Table S2.** Suggestive SNPs associated with Aβ positivity. **Table S3.** Association of genome-wide suggestive SNPs (*p*<1.0×10^-6^ ) with Aβ positivity based on SUVR. **Table S4.** Association of previously reported Aβ risk loci from European populations with Aβ positivity in the Korean population.

**Additional file 2: Figure S1.** Histogram of t-values obtained from the permutations. Red dotted lines indicate the lowest 5% of the 10,000 permutations. Red arrows indicate the observed t-value obtained from the original dataset.

## Data Availability

The datasets used and analyzed during the current study are available from the corresponding author on reasonable request.

## Abbreviations

Aβ: Amyloid-beta
AD: Alzheimer’s disease
ADD: Alzheimer’s disease dementia
aMCI: Amnestic mild cognitive impairment
AUC: Area under curve
BICWALZS: Biobank Innovation for chronic Cerebrovascular disease With ALZheimer’s disease Study
CCDC146: Coiled-coil domain containing the 146
CI: Confidence interval
cis-eQTL: Cis-expression quantitative trait loci
CU: Cognitive unimpaired
FGL2: Fibrinogen-like protein 2
GWAS: Genome-wide association studies
MAF: Minor allele frequency
MNI: Montreal Neurological Institute
NES: Normalized effect size
OR: Odds ratio
PC: Principal component
PET: Positron emission tomography
SNP: Single nucleotide polymorphism
KBASE-V: Korean Brain Aging Study for the Early Diagnosis and Prediction of AD
