# Supplementary material for: Identifying novel genetic variants for brain amyloid deposition: a genome-wide association study in the Korean population
Source: Alzheimers Res Ther. 2021 Jun 21;13:117. doi: 10.1186/s13195-021-00854-z (PMC8215820; doi:10.1186/s13195-021-00854-z)
Supplement: Supplementary file 1 — Additional file 1: Table S1. Significant (p value< 5.0×10^-8^) SNPs associated with Aβ positivity. Table S2. Suggestive SNPs associated with Aβ positivity. Table S3. Association of genome-wide suggestive SNPs (p<1.0×10^-6^ ) with Aβ positivity based on SUVR. Table S4. Association of previously reported Aβ risk loci from European populations with Aβ positivity in the Korean population. [file 13195_2021_854_MOESM1_ESM.docx]

**Supplementary Table**

**Table S1. Significant (*p* value< 5.0×10^-8^) SNPs associated with Aβ positivity**

| **SNP** | **CHR** | **BP** | **Effective allele** | **OR** | ***p*** | ***p*^†^** |
| --- | --- | --- | --- | --- | --- | --- |
| rs8112196 | 19 | 45325309 | A | 0.632 | 9.12×10^-8^ | 0.086 |
| rs6509172 | 19 | 45325391 | T | 0.616 | 7.06×10^-9^ | 0.044 |
| rs1871045 | 19 | 45326768 | T | 0.613 | 6.32×10^-9^ | 0.056 |
| rs73050216 | 19 | 45367502 | C | 0.527 | 1.93×10^-14^ | 0.049 |
| rs41290098 | 19 | 45370278 | T | 0.547 | 4.59×10^-13^ | 0.165 |
| rs12610605 | 19 | 45370838 | G | 1.917 | 1.21×10^-14^ | 0.354 |
| rs404935 | 19 | 45372794 | A | 3.472 | 1.07×10^-29^ | 0.193 |
| rs395908 | 19 | 45373565 | A | 3.332 | 2.85×10^-28^ | 0.451 |
| rs34278513 | 19 | 45378144 | T | 2.868 | 1.12×10^-21^ | 0.515 |
| rs412776 | 19 | 45379516 | A | 3.601 | 5.02×10^-31^ | 0.149 |
| rs3865427 | 19 | 45380961 | A | 3.122 | 5.34×10^-24^ | 0.314 |
| rs6859 | 19 | 45382034 | A | 2.110 | 1.39×10^-17^ | 0.654 |
| rs3852860 | 19 | 45382966 | C | 2.875 | 5.43×10^-27^ | 0.113 |
| rs3852861 | 19 | 45383061 | G | 2.850 | 1.19×10^-26^ | 0.135 |
| rs71352237 | 19 | 45383079 | C | 3.288 | 9.49×10^-25^ | 0.493 |
| rs34224078 | 19 | 45383115 | G | 3.288 | 9.49×10^-25^ | 0.493 |
| rs35879138 | 19 | 45383139 | A | 3.307 | 6.24×10^-25^ | 0.430 |
| rs166907 | 19 | 45386855 | G | 6.140 | 3.09×10^-8^ | 0.231 |
| rs12972156 | 19 | 45387459 | G | 3.830 | 7.66×10^-29^ | 0.427 |
| rs12972970 | 19 | 45387596 | A | 3.830 | 7.66×10^-29^ | 0.427 |
| rs34342646 | 19 | 45388130 | A | 3.830 | 7.66×10^-29^ | 0.427 |
| rs283815 | 19 | 45390333 | G | 3.338 | 6.85×10^-32^ | 0.298 |
| rs6857 | 19 | 45392254 | T | 3.681 | 5.38×10^-28^ | 0.261 |
| rs71352238 | 19 | 45394336 | C | 3.748 | 2.03×10^-28^ | 0.368 |
| rs184017 | 19 | 45394969 | G | 3.289 | 2.24×10^-31^ | 0.396 |
| rs157580 | 19 | 45395266 | G | 0.462 | 2.12×10^-19^ | 0.154 |
| rs2075650 | 19 | 45395619 | G | 3.748 | 2.03×10^-28^ | 0.368 |
| rs157581 | 19 | 45395714 | C | 2.820 | 3.31×10^-27^ | 0.966 |
| rs34404554 | 19 | 45395909 | G | 3.716 | 3.22×10^-28^ | 0.293 |
| rs11556505 | 19 | 45396144 | T | 3.716 | 3.22×10^-28^ | 0.293 |
| rs157582 | 19 | 45396219 | T | 3.326 | 8.93×10^-32^ | 0.373 |
| rs59007384 | 19 | 45396665 | T | 2.802 | 3.38×10^-23^ | 0.965 |
| rs157583 | 19 | 45396673 | T | 6.677 | 5.65×10^-9^ | 0.117 |
| rs157587 | 19 | 45398206 | G | 6.571 | 7.71×10^-9^ | 0.122 |
| rs205909 | 19 | 45400775 | G | 6.677 | 5.65×10^-9^ | 0.117 |
| rs491153 | 19 | 45402368 | T | 6.677 | 5.65×10^-9^ | 0.117 |
| rs490243 | 19 | 45402470 | T | 6.677 | 5.65×10^-9^ | 0.117 |
| rs417357 | 19 | 45403119 | T | 6.775 | 4.24×10^-9^ | 0.114 |
| rs394819 | 19 | 45404579 | T | 6.775 | 4.24×10^-9^ | 0.114 |
| rs405697 | 19 | 45404691 | G | 2.299 | 1.86×10^-21^ | 0.170 |
| rs10119 | 19 | 45406673 | A | 4.948 | 1.21×10^-40^ | 0.967 |
| rs435380 | 19 | 45407118 | A | 6.576 | 7.63×10^-9^ | 0.133 |
| rs446037 | 19 | 45407437 | T | 6.577 | 7.59×10^-9^ | 0.147 |
| rs434132 | 19 | 45407720 | G | 6.577 | 7.59×10^-9^ | 0.147 |
| rs439382 | 19 | 45408475 | G | 6.573 | 7.68×10^-0^ | 0.107 |
| rs440446 | 19 | 45409167 | G | 2.318 | 3.06×10^-22^ | 0.318 |
| rs769449 | 19 | 45410002 | A | 4.601 | 4.83×10^-33^ | 0.223 |
| rs429358 | 19 | 45411941 | C | 5.275 | 9.03×10^-42^ | 0.601 |
| rs75627662 | 19 | 45413576 | T | 3.392 | 1.55×10^-28^ | 0.790 |
| rs439401 | 19 | 45414451 | C | 2.295 | 5.58×10^-22^ | 0.279 |
| rs10414043 | 19 | 45415713 | A | 4.285 | 8.20×10^-32^ | 0.216 |
| rs7256200 | 19 | 45415935 | T | 4.285 | 8.20×10^-32^ | 0.216 |
| rs483082 | 19 | 45416178 | T | 4.024 | 1.01×10^-37^ | 0.213 |
| rs584007 | 19 | 45416478 | G | 2.284 | 8.19×10^-22^ | 0.310 |
| rs438811 | 19 | 45416741 | T | 4.024 | 1.01×10^-37^ | 0.213 |
| rs5117 | 19 | 45418790 | C | 3.316 | 1.17×10^-27^ | 0.921 |
| rs12721046 | 19 | 45421254 | A | 4.497 | 6.23×10^-35^ | 0.209 |
| rs12721051 | 19 | 45422160 | G | 4.493 | 5.81×10^-35^ | 0.214 |
| rs56131196 | 19 | 45422846 | A | 4.517 | 3.73×10^-35^ | 0.291 |
| rs4420638 | 19 | 45422946 | G | 4.517 | 3.73×10^-35^ | 0.291 |
| rs157595 | 19 | 45425460 | G | 2.197 | 4.31×10^-20^ | 0.520 |

*P* value was calculated using logistic regression analysis. ^†^*P* value was calculated using logistic regression analysis with adjustment of the *APOE* ɛ4 genotype.

Abbreviation: A = adenine; C = cytosine; G = guanine; T = thymine; CHR = chromosome; BP = base pair; OR = odds ratio; SNP = single nucleotide polymorphism

**Table S2. Suggestive SNPs associated with Aβ positivity**

| **SNP** | **CHR** | **BP** | **Effective allele** | **OR** | ***p*** |
| --- | --- | --- | --- | --- | --- |
| ***p-*value < 1.0×10^-6^** | | | | | |
| rs73375428 | 7 | 76907550 | G | 0.519 | 2.71×10^-7^ |
| rs6978259 | 7 | 76909167 | C | 0.522 | 4.62×10^-7^ |
| rs2903923 | 7 | 76907750 | G | 0.529 | 5.15×10^-7^ |
| rs3828947 | 7 | 76908199 | C | 0.529 | 5.15×10^-7^ |
| rs6958464 | 7 | 76909035 | T | 0.526 | 6.28×10^-7^ |
| rs11983537 | 7 | 76908690 | T | 0.558 | 7.58×10^-7^ |
| rs112599253 | 7 | 76931677 | T | 0.561 | 1.56×10^-7^ |
| rs79761449 | 7 | 76925493 | T | 0.564 | 2.50×10^-7^ |
| rs6971106 | 7 | 76929191 | T | 0.564 | 2.50×10^-7^ |
| **1.0×10^-6^ < *p-*value < 1.0×10^-5^** | | | | | |
| rs113931965 | 7 | 76930080 | T | 0.564 | 2.50×10^-6^ |
| rs113014884 | 7 | 76930314 | C | 0.564 | 2.50×10^-6^ |
| rs58731747 | 7 | 76935492 | A | 0.568 | 2.75×10^-6^ |
| rs1077236 | 8 | 130640501 | T | 1.612 | 2.85×10^-6^ |
| rs10092679 | 8 | 130682158 | T | 1.580 | 5.33×10^-6^ |
| rs1196470 | 1 | 151706235 | A | 0.669 | 5.43×10^-6^ |
| rs4872051 | 8 | 22945345 | G | 0.687 | 5.66×10^-6^ |
| rs140005 | 22 | 36943544 | A | 1.488 | 5.76×10^-6^ |
| rs140008 | 22 | 36944165 | T | 1.488 | 5.76×10^-6^ |
| rs140010 | 22 | 36944356 | C | 1.488 | 5.76×10^-6^ |
| rs68055908 | 8 | 130667115 | T | 1.579 | 5.85×10^-6^ |
| rs59674745 | 7 | 76913192 | C | 0.511 | 5.86×10^-6^ |
| rs6470745 | 8 | 130641921 | G | 1.576 | 6.36×10^-6^ |
| rs6985032 | 8 | 130671748 | T | 1.561 | 7.46×10^-6^ |
| rs4295627 | 8 | 130685457 | G | 1.569 | 7.61×10^-6^ |
| rs28572791 | 8 | 130641782 | C | 1.569 | 7.92×10^-6^ |
| rs2868782 | 7 | 76907862 | A | 0.517 | 8.06×10^-6^ |
| rs7306151 | 12 | 110092612 | C | 0.619 | 8.24×10^-6^ |
| rs6970348 | 7 | 76929400 | A | 0.610 | 8.27×10^-6^ |
| rs140018 | 22 | 36946330 | C | 1.479 | 8.97×10^-6^ |
| rs140019 | 22 | 36946491 | G | 1.479 | 8.97×10^-6^ |
| rs140011 | 22 | 36945022 | G | 1.479 | 9.02×10^-6^ |
| rs140012 | 22 | 36945289 | C | 1.479 | 9.02×10^-6^ |
| rs140014 | 22 | 36945641 | C | 1.479 | 9.02×10^-6^ |
| rs140016 | 22 | 36945847 | C | 1.479 | 9.02×10^-6^ |
| rs140017 | 22 | 36945947 | A | 1.479 | 9.02×10^-6^ |
| rs144786745 | 8 | 130658990 | T | 1.569 | 9.23×10^-6^ |
| rs4766712 | 12 | 114491139 | T | 1.511 | 9.40×10^-6^ |
| rs5022680 | 8 | 130685516 | T | 1.562 | 9.42×10^-6^ |

*P*-value and odds ratio were calculated using logistic regression analysis.

Abbreviations: A = adenine; C = cytosine; G = guanine; T = thymine; CHR = chromosome; BP = base pair; OR = odds ratio; SNP = single nucleotide polymorphism

**Table S3. Association of genome-wide suggestive SNPs (*p*<1.0×10^-6^ ) with Aβ positivity based on SUVR**

|  |  | **Analysis 1** | | | |  | **Analysis 2** | | | |
| --- | --- | --- | --- | --- | --- | --- | --- | --- | --- | --- |
|  |  | **Discovery data (n=824)** | | **Replication data (n=260)** | |  | **Discovery data (n=824)** | | **Replication data (n=260)** | |
| **SNP** | **EA** | **OR** | ***p*** | **OR** | ***p*** |  | **OR** | ***p*** | **OR** | ***p*** |
| **rs73375428** | G | 0.608 | 0.00098 | 0.551 | 0.047 |  | 0.644 | 0.00842 | 0.438 | 0.018 |
| **rs2903923** | G | 0.609 | 0.00098 | 0.548 | 0.042 |  | 0.644 | 0.00832 | 0.438 | 0.017 |
| **rs3828947** | C | 0.609 | 0.00098 | 0.548 | 0.042 |  | 0.644 | 0.00832 | 0.448 | 0.017 |
| **rs11983537** | T | 0.621 | 0.00087 | 0.557 | 0.041 |  | 0.656 | 0.00777 | 0.471 | 0.023 |
| **rs112599253** | T | 0.696 | 0.0116 | 0.707 | 0.201 |  | 0.738 | 0.0554 | 0.642 | 0.160 |
| **rs79761449** | T | 0.680 | 0.00787 | 0.707 | 0.201 |  | 0.710 | 0.0323 | 0.642 | 0.160 |
| **rs6971106** | T | 0.680 | 0.00787 | 0.707 | 0.201 |  | 0.710 | 0.0323 | 0.642 | 0.160 |
| **rs6978259** | C | 0.614 | 0.00151 | 0.656 | 0.168 |  | 0.662 | 0.0156 | 0.555 | 0.090 |
| **rs6958464** | T | 0.614 | 0.00151 | 0.634 | 0.143 |  | 0.662 | 0.0156 | 0.510 | 0.059 |

Analysis 1 is a logistic regression analysis, expressed as Aβ positivity (defined by SUVR) = β_0_ + β_1_ age + β_2_ sex + β_3_ PC_1_ + β_4_ PC_2_ + β_5_ PC_3_ + β_6_ SNP

Analysis 2 is a logistic regression analysis, expressed as Aβ positivity (defined by SUVR) = β_0_ + β_1_ age + β_2_ sex + β_3_ PC_1_ + β_4_ PC_2_ + β_5_ PC_3_ + β_6_ *APOE* ɛ4+ β_7_ SNP

Abbreviations: EA, effective allele; OR, odds ratio; SNP, single nucleotide polymorphism

**Table S4. Association of previously reported Aβ risk loci from European populations with Aβ positivity in the Korean population**

| **SNP** | **CHR** | **BP** | **Closest Gene** | **EA** | **European (Yan Q *et al*)** | | | **Korean (our cohort)** | | | **East Asian** |
| --- | --- | --- | --- | --- | --- | --- | --- | --- | --- | --- | --- |
|  |  |  |  |  | **OR^a^** | ***p*^†^** | **EAF^¶^** | **OR^††^** | ***p*^††^** | **EAF^§^** | **EAF^¶^** |
| rs429358 | 19 | 45411941 | *APOE* | C | 1.19 | 9.0×10^-30^ | 0.155 | 5.27 | 9.0×10^-42^ | 0.125 | 0.086 |
| rs13260032 | 8 | 132451455 | *ADCY8, EFR3A* | C | 0.92 | 4.8×10^-7^ | 0.449 | NA | NA | NA | 0.511 |
| rs4680057 | 3 | 153096985 | *RAP2B, C3orf79* | A | 1.06 | 9.6×10^-7^ | 0.446 | 1.09 | 0.274 | 0.466 | 0.411 |
| rs12908891 | 15 | 64236441 | *DAPK2* | G | 1.06 | 1.3×10^-6^ | 0.506 | 0.90 | 0.463 | 0.114 | 0.075 |
| rs7377304 | 4 | 187129780 | *CYP4V2* | T | 0.94 | 2.4×10^-6^ | 0.528 | 0.94 | 0.518 | 0.36 | 0.317 |
| rs55708341 | 21 | 45627581 | *C21orf33,ICOSLG* | T | 1.07 | 2.5×10^-6^ | 0.185 | 0.90 | 0.351 | 0.191 | 0.223 |
| rs9831119 | 3 | 84712077 | *LINC00971* | C | 0.92 | 2.9×10^-6^ | 0.094 | 0.93 | 0.385 | 0.416 | 0.304 |
| rs9531483 | 13 | 84244873 | *SLITRK1* | A | 0.94 | 3.6×10^-6^ | 0.279 | 1.09 | 0.289 | 0.406 | 0.428 |
| rs6722000 | 2 | 209075957 | *C2orf80,IDH1* | G | 1.07 | 4.9×10^-6^ | 0.209 | 0.94 | 0.882 | 0.015 | 0.029 |
| rs11923588 | 3 | 184459667 | *MAGEF1,LOC101928992* | T | 0.83 | 5.6×10^-6^ | 0.062 | 0.81 | 0.058 | 0.159 | 0.202 |
| rs66837203 | 4 | 36897136 | *DTHD1,MIR4801* | T | 1.11 | 6.0×10^-6^ | 0.050 | 1.01 | 0.844 | 0.252 | 0.304 |
| rs200028958 | 4 | 70923661 | *HTN1* | A | 1.10 | 6.2×10^-6^ | 0.110 | NA | NA | NA | 0.199 |
| rs4526799 | 12 | 57280586 | *HSD17B6,SDR9C7* | T | 0.95 | 7.2×10^-6^ | 0.348 | NA | NA | NA | 0.568 |
| rs17105538 | 1 | 81315043 | *ELTD1,LPHN2* | G | 1.08 | 7.6×10^-6^ | 0.122 | 0.96 | 0.854 | 0.045 | 0.033 |
| rs62121100 | 2 | 3093952 | *LINC01250* | G | 0.93 | 8.4×10^-6^ | 0.141 | 1.02 | 0.842 | 0.083 | 0.111 |
| rs1809136 | 2 | 11152180 | *KCNF1,FLJ33534* | G | 1.17 | 9.9×10^-6^ | 0.084 | 0.92 | 0.340 | 0.366 | 0.453 |

^†^Odds ratios and *p* values were reported in a previous study [1]. Among the 16 Aβ associated SNPs reported by Yan et al [1], 13 SNPs were available in our data after QC. ^††^Odds ratio and *p* value were calculated in our cohort using logistic regression analysis. ^¶^EAF is based on the 1000 Genomes Project phase 3 data according to ethnicities (European and East Asians) [2]. ^§^EAF is based on our cohort (Korean).

Abbreviation: A = adenine; C = cytosine; G = guanine; T = thymine; CHR = chromosome; BP = base pair; EA = effective allele; NA = not applicable; EAF = effective allele frequency; OR = odds ratio; SNP = single nucleotide polymorphism


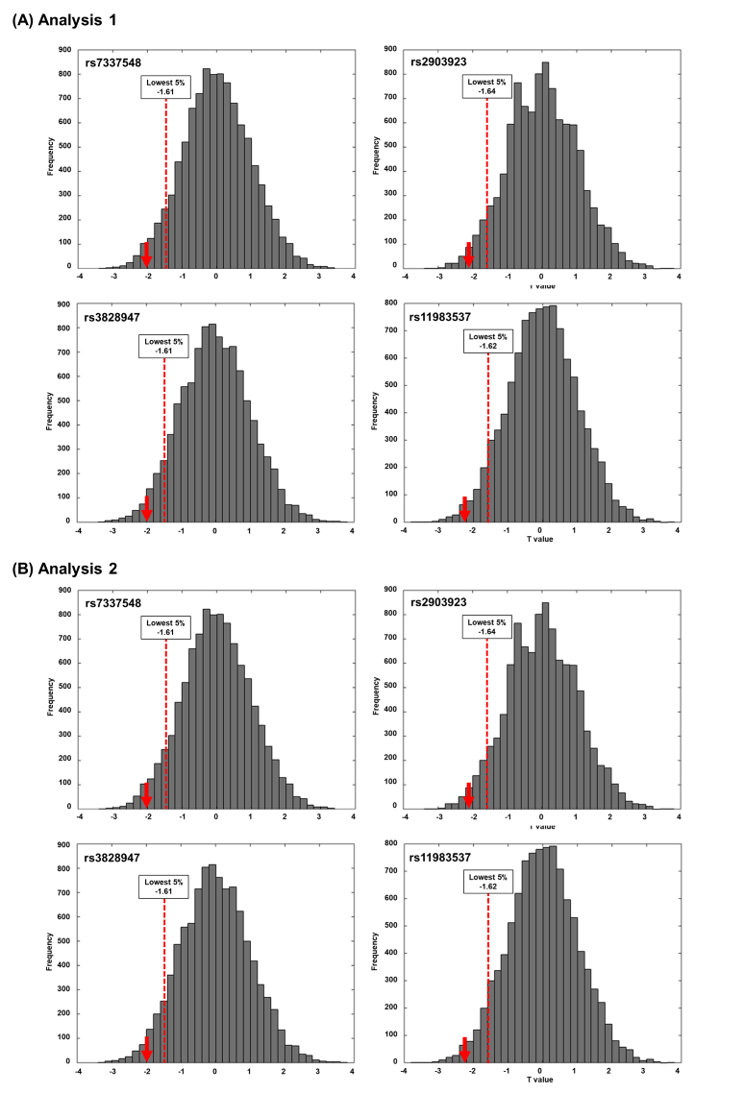


**Figure S1.** Histogram of t-values obtained from the permutations. Red dotted lines indicate the lowest 5% of the 10,000 permutations. Red arrows indicate the observed t-value obtained from the original dataset.

Analysis 1 is a logistic regression analysis, expressed as Aβ positivity= β_0_ + β_1_ age + β_2_ sex + β_3_ SNP.

Analysis 2 is a logistic regression analysis, expressed as Aβ positivity= β_0_ + β_1_ age + β_2_ sex + β_3_ *APOE* ɛ4+ β_4_ SNP.

**Supplementary references**

1. Yan Q, Nho K, Del-Aguila JL, et al. Genome-wide association study of brain amyloid deposition as measured by Pittsburgh Compound-B (PiB)-PET imaging. Mol Psychiatry. 2018:1-13.

2. Consortium GP. A global reference for human genetic variation. Nature 2015;526:68-74.
