# Supplementary figures and images for: Identifying novel genetic variants for brain amyloid deposition: a genome-wide association study in the Korean population
Source: Alzheimers Res Ther. 2021 Jun 21;13:117. doi: 10.1186/s13195-021-00854-z (PMC8215820; doi:10.1186/s13195-021-00854-z)

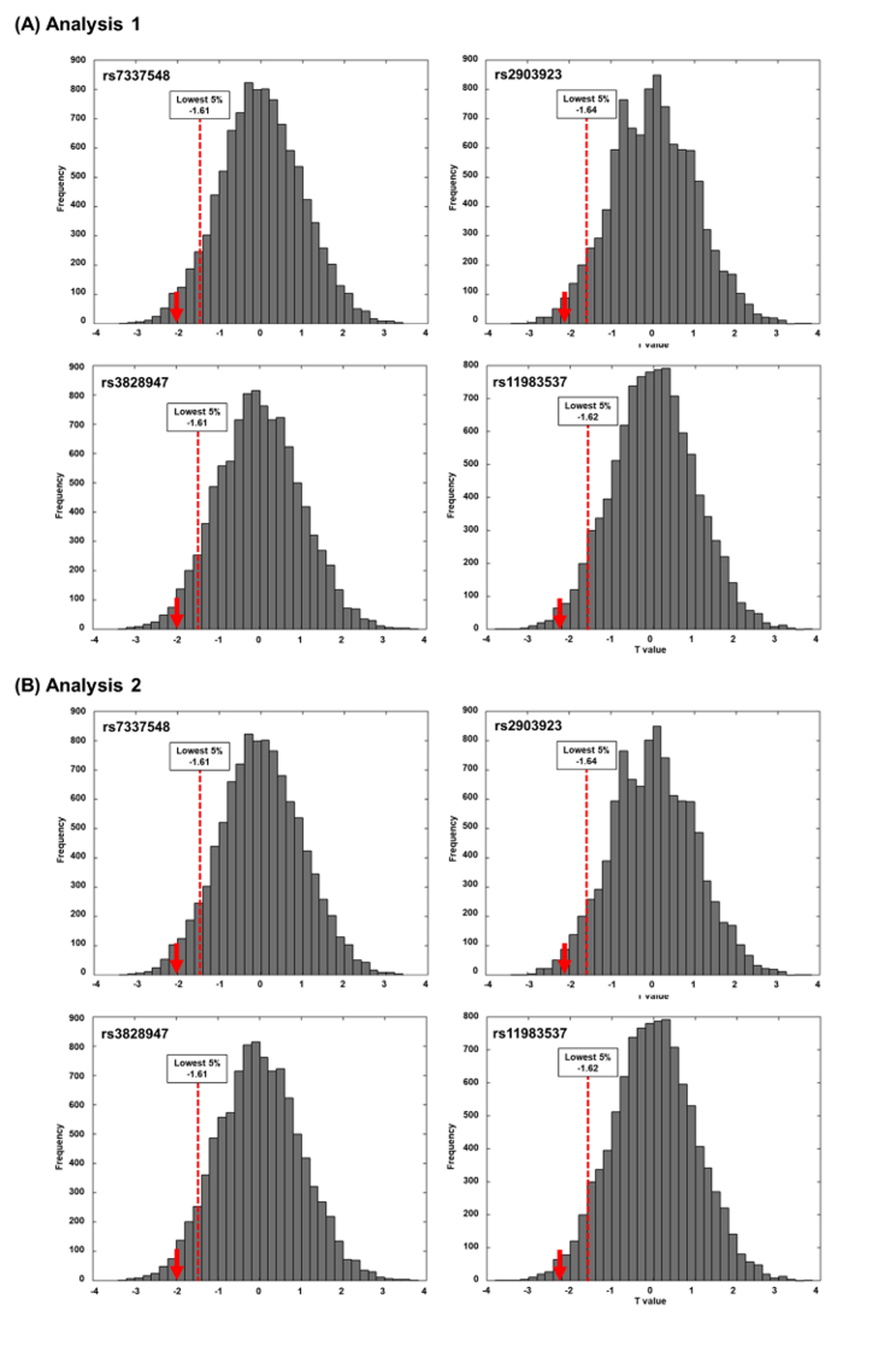

Supplement: Supplementary file 2 — Additional file 2: Figure S1. Histogram of t-values obtained from the permutations. Red dotted lines indicate the lowest 5% of the 10,000 permutations. Red arrows indicate the observed t-value obtained from the original dataset. [file 13195_2021_854_MOESM2_ESM.tiff]
